# Framework for community functioning: synthesis of stress gradient and resource partitioning concepts

**DOI:** 10.7717/peerj.3885

**Published:** 2017-10-02

**Authors:** Sophia I. Passy

**Affiliations:** Department of Biology, University of Texas at Arlington, Arlington, TX, United States of America

**Keywords:** Competition, Biodiversity-ecosystem functioning, Facilitation, Nitrogen fixers, Stress gradient hypothesis, Resource partitioning, Tradeoffs, Diatom guilds, Streams

## Abstract

To understand how communities function and generate abundance, I develop a framework integrating elements from the stress gradient and resource partitioning concepts. The framework suggests that guild abundance depends on environmental and spatial factors but also on inter-guild interactions (competitor or facilitator richness), which can alter the fundamental niche of constituent species in negative (competition) or positive direction (facilitation). Consequently, the environmental and spatial mechanisms driving guild abundance would differ across guilds and interaction modes. Using continental data on stream diatoms and physico-chemistry, the roles of these mechanisms were tested under three interaction modes—shared preference, distinct preference, and facilitative, whereby pairs of guilds exhibited, respectively, a dominance-tolerance tradeoff along a eutrophication gradient, specialization along a pH gradient, or a donor-recipient relationship along a nitrogen gradient. Representative of the shared preference mode were the motile (dominant) and low profile (tolerant) guilds, of the distinct preference mode—the acidophilous and alkaliphilous (low profile) guilds, and of the facilitative mode—nitrogen fixers (donors) and motile species (recipients). In each mode, the influences of environment, space (latitude and longitude), and competitor or facilitator richness on guild density were assessed by variance partitioning. Pure environment constrained most strongly the density of the dominant, the acidophilous, and the recipient guild in the shared preference, distinct preference, and facilitative mode, respectively, while spatial effects were important only for the low profile guild. Higher competitor richness was associated with lower density of the tolerant guild in the shared preference mode, both guilds in the distinct preference mode, and the donor guild in the facilitative mode. Conversely, recipient density in the facilitative mode increased with donor richness in stressful nitrogen-poor environments. Thus, diatom guild abundance patterns were determined primarily by biotic and/or environmental impacts and, with the exception of the low profile guild, were insensitive to spatial effects. This framework identifies major sources of variability in diatom guild abundance with implications for the understanding of biodiversity-ecosystem functioning.

## Introduction

### Background

An important question in ecology with implications for community functioning and conservation is what drives the abundance variability in species communities. The proposed core mechanisms are few, i.e., species differential abilities to cope with the environment, to disperse, and to interact with one another. Nevertheless, our current understanding of the relative roles of these mechanisms and their interactive effects is still inadequate. To address this problem, I develop a framework, which integrates elements from the stress gradient hypothesis (Bertness & Callaway, 1994) and the resource partitioning theory (Rosenzweig, 1991; Wisheu, 1998), and predicts diatom guild abundance along environmental, spatial, and biotic gradients in the freshwater metacommunity.

The stress gradient hypothesis contends that negative interactions prevail in low stress environments, while positive intra- and interspecific interactions are more prominent under stressful conditions where there are clear benefits of growing in a group of conspecifics or in the presence of heterospecifics (Bertness & Callaway, 1994). For example, in the high intertidal zone a solitary alga will die of desiccation, while the population will endure; in arid zones understory plants survive only under the canopy of a nurse plant (He & Bertness, 2014). Thus, facilitation, which has been globally documented (He, Bertness & Altieri, 2013), increases the realized niche beyond the boundaries of the fundamental niche (Bruno, Stachowicz & Bertness, 2003), allows species survival and coexistence, and deserves a more prominent place in metacommunity research.

The resource partitioning perspective relates coexistence of competing species to specific tradeoffs along environmental gradients (Rosenzweig, 1991; Wisheu, 1998; McGill et al., 2006). There are two major competitive scenarios—a distinct preference and a shared preference (Rosenzweig, 1991). In the distinct preference mode, species have unique optima along an environmental gradient but overlapping tolerances (the spread around the optimum). Consequently, to avoid competition, these species occupy the fraction of their fundamental niche where they perform the best and have the highest fitness. Contrariwise, species in the shared preference mode have similar optima, e.g., in resource-replete, disturbance-free habitats, but they segregate along environmental gradients due to a dominance-tolerance tradeoff. Species invest in either acquiring high quality resources or tolerating unfavorable conditions. As a result, dominant species inhabit the optimal conditions, while the realized niche of the tolerant species is restricted to suboptimal environments.

### The proposed framework

The premise of this framework is that a community function, i.e., diatom guild abundance accumulation, is governed by a specific environmental gradient, spatial distribution with a potential impact on dispersal, and local biotic interactions in characteristic and predictable ways. The environmental conditions control species growth rates either by providing subsidy (e.g., resource supply) or imposing stress, as in the case of regulatory gradients (e.g., pH). Notably, the environment (e.g., current velocity) can also influence the rates of immigration and emigration but due to the lack of data, such gradients are not considered here.

**Figure 1:**
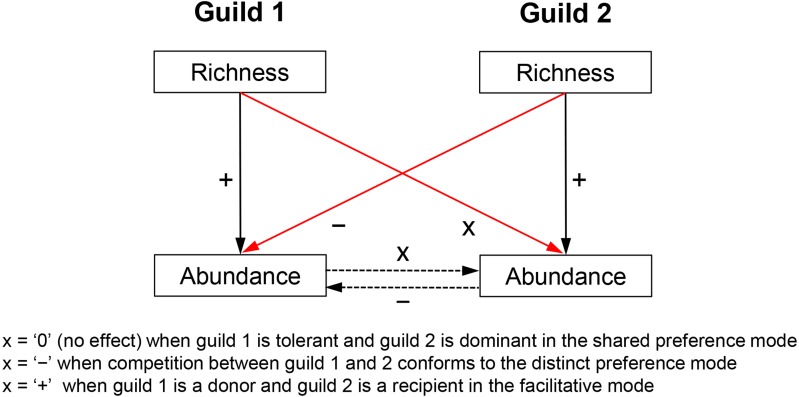
Hypothesized relationships of guild richness and abundance in guilds involved in competitive vs. facilitative interactions. Richness determines the abundance of a guild, which in turn may affect the abundance of a competitor or a recipient guild (dashed arrows), i.e., through respectively consumption or production of a limiting resource (e.g., bioavailable nitrogen). Therefore, the richness of a guild has an influence on the abundance of another guild (red arrows) and can be used as a proxy measure of competition (negative effect) or facilitation (positive effect). Richness of an inferior competitor, i.e., the tolerant guild in the shared preference mode, is expected to have little to no effect on the abundance of a superior competitor, i.e., the dominant guild (see Fig. 2 for more details on specific guilds and tradeoffs). The positive effect of richness on guild abundance (solid black arrows) was confirmed with a correlation analysis, i.e., Pearson *r* ranged between 0.18 and 0.48 (*P* < 0.05) for the guilds in the three studied modes.

Biotic effects determine the realized niche of a response guild and, therefore, have an independent effect on its abundance variability across communities. Biotic effects too constrain growth rates, e.g., by reducing (competition) or increasing resource uptake (facilitation). Competition can also limit immigration by controlling the available space but such influences are of much lesser importance in the multilayered biofilms studied here. Admittedly, predators can mediate coexistence of competitors (Shurin & Allen, 2001), but due to the absence of data, this question is not addressed here. Competitive/facilitative effects are difficult to estimate without experimentation but the richness of a competitor/facilitator guild is deemed here a suitable proxy measure for the following reasons. Species richness is broadly shown to be a positive predictor of abundance (Hooper et al., 2005; Cardinale et al., 2012). This means that a species-rich competitor or facilitator guild would generate greater abundance and/or utilize resources more efficiently and would, therefore, have a stronger impact on its target guild. This impact includes consumption of shared limiting resources in the case of competition (e.g., nitrogen and phosphorus) or production of a limiting resource in the case of facilitation (e.g., synthesis of ammonia by nitrogen fixers, which can be used by non-nitrogen fixers). Thus, species richness of a guild can indirectly control the abundance of another guild (Fig. 1)—a notion later confirmed by regression analyses.

**Figure 2:**
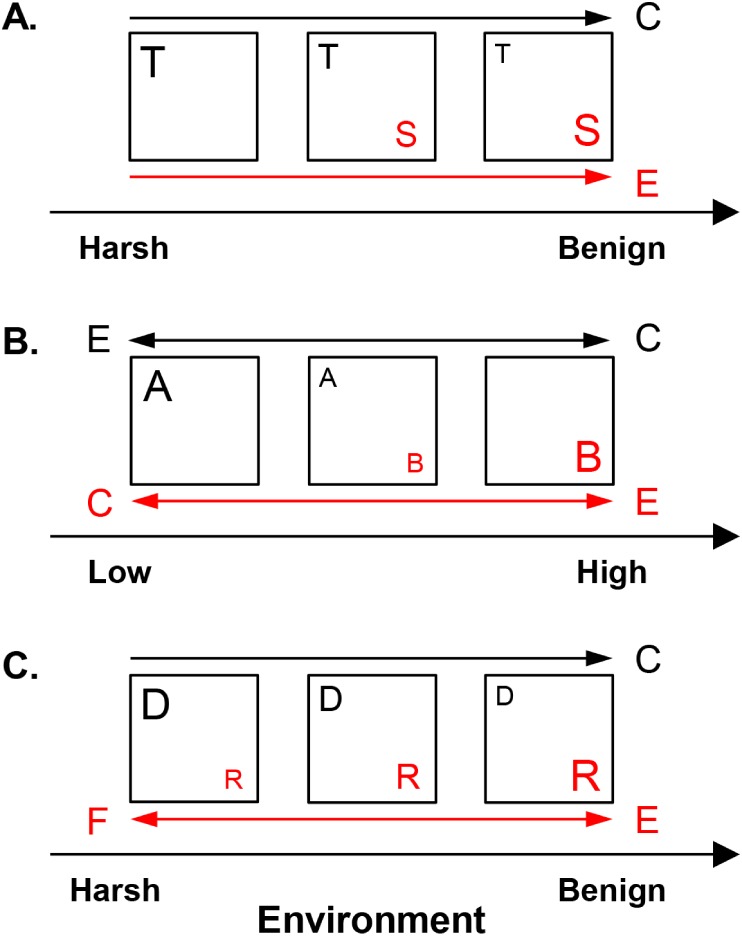
Hypothesized metacommunity processes, operating in competitive and facilitative systems along environmental gradients. (A) *Shared preference mode*. Species invest in either tolerance to harsh conditions, such as low resource availability (guild T, represented here by the low profile diatom guild) or dominance in benign environments, such as high resource supply (guild S, represented here by the motile diatom guild). (B) *Distinct preference mode.* Guilds A and B have overlapping distributions but distinct optima along an environmental gradient at low vs. high levels, respectively. Acidophilous and alkaliphilous diatoms with preference for pH <  7 vs. pH ≥ 7, respectively, exemplify these guilds. (C) *Facilitative mode.* A guild provides (donor, D) or receives benefits (recipient, R) under harsh conditions, e.g., low nitrogen concentrations. In benign environments, e.g., high nitrogen levels, the competitively superior recipient suppresses the donor. Nitrogen fixing and eutrophic non-nitrogen fixing diatoms represent the donor and the recipient guild, respectively. Squares represent communities. The size of the letter denoting a guild corresponds to its abundance. The drivers of guild abundance are environmental (E) and biotic, including competition (C) and facilitation (F). Guilds are given in red or black and this color scheme is maintained for the arrows, which point in the direction of increase of a given driver for the respective guild.

The present work outlines three non-exclusive interaction modes—shared preference, distinct preference, and facilitative, which determine the abundance of the constituent guilds in response to environmental and biotic factors (Fig. 2). In the shared preference mode, tolerant and sensitive (dominant) guilds (guilds T and S, respectively in Fig. 2A) segregate along an environmental gradient as a result of (i) competitive suppression of tolerant species by dominant forms in optimal conditions and (ii) inability of dominant species to survive in unfavorable environments (Wisheu, 1998). Consequently, it is expected the abundance of tolerant species to be driven primarily by competition, but only in environments suitable for their competitor (Fig. 2A). Since tolerant species can inhabit the entire environmental gradient, their abundance would show weak response to environmental factors when the effect of competitors is controlled for. Conversely, the abundance of the dominant guild, capable of positive growth only under restricted environmental conditions, is expected to be governed mainly by the environment (Table 1, Fig. 2A).

**Table 1:** Major hypothesized and observed (Fig. 5) drivers of guild density across interaction modes with corresponding variance partitioning terms, given in parentheses.

Mode	Guild	Drivers (Variance partitioning term)	
		Hypothesized	Observed (adjusted *R*^2^ ≥ 0.05)
**Shared preference**	Tolerant (e.g., Low profile guild)	GR	GR ([GR_S_|E,S])
	Sensitive (e.g., Motile guild)	E	E
**Distinct preference**	Guild A (e.g., Acidophilous guild)	E, GR	E ([E|GR_B_])
	Guild B (e.g., Alkaliphilous guild)	E, GR	E+GR ([E∩GR_A_|S]), S ([S|E, GR_A_])
**Facilitative**	Donor (e.g., N_2_ fixer guild)	GR	GR_R_
	Recipient (e.g., Motile guild)	E, GR	E ([E|GR_D_]), GR ([GR_D_|E])

**Notes.**

E environment S space GR guild richness GR_T_, GR_S_, GR_A_, GR_B_, GR_D_, and GR_R_ richness of tolerant, sensitive, A-, B-, donor, and recipient guilds, respectively

**Figure 3:**
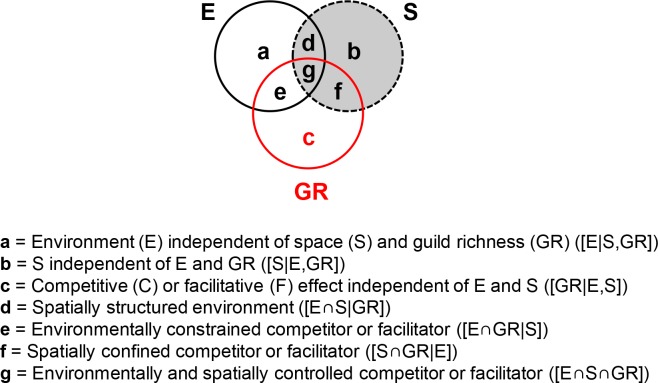
General variance partitioning model giving the fractions of explained variance (**a**–**g**) in the dependent variable (i.e., diatom guild density here).

The variance partitioning fractions are given in Fig. 3.

In the distinct preference mode (Fig. 2B), guilds exhibit differential environmental preferences, each one reaching maximum abundance around its environmental optimum and declining away from this optimum because of increasing environmental unsuitability and competition (Wisheu, 1998). In Fig. 2B, guilds A and B have respective optima at low vs. high values of an environmental gradient, e.g., acidophilous and alkaliphilous species with pH optima <7 and ≥7, respectively. Thus, abundance of both guilds is envisioned to be driven by the environment, while competition may become important in the region of distributional overlap.

In the facilitative mode, species are defined as donors (guild D in Fig. 2C) or recipients (guild R in Fig. 2C), e.g., nitrogen fixers vs. non-nitrogen fixers, respectively. Donors (facilitators) thrive under harsh conditions and through environmental amelioration support the recipients, which may not survive in their absence (Bertness & Callaway, 1994). However, donors are inferior competitors in favorable environments where they may be excluded by the competitively superior recipients (Bertness & Shumway, 1993). Thus, in environments suitable for recipients, donor abundance would decline due to competition. Recipient abundance would respond positively to donors (facilitative control) in harsh conditions and to environmental favorability (Fig. 2C).

Diatom guilds are ubiquitous with comparatively low biogeographic fidelity, suggesting that their composition may be shaped by weak historic effects (Soininen et al., 2016). Regional scale mass effects across lower stream reaches, on the other hand, have been documented to constrain the composition of diatom communities and certain diatom guilds (Jamoneau et al., 2017). Therefore, it is possible that guild abundance is subjected to mass effects, whereby species populations in unfavorable habitats of high environmental stress or competition are maintained via immigration from favorable source localities. Furthermore, at high dispersal rates, a greater proportion of the regional species pool can reach a particular locality, increasing the richness of resident guilds, and consequently, strengthening their interactions, as suggested in Fig. 1.

This study tests the following two hypotheses. Hypothesis 1: competitor or facilitator richness would impact guild abundance in addition to the environment, given that the fundamental niche of a guild is reduced by competition (Rosenzweig, 1991; Wisheu, 1998; McGill et al., 2006) or increased by facilitation (Bruno, Stachowicz & Bertness, 2003). Hypothesis 2: the effects of the environment and competitor/facilitator richness on guild abundance would depend on traits and interaction mode, as specified in the proposed framework (Fig. 2), but these effects may also exhibit spatial dependence.

### Testing the proposed framework

Variance partitioning is a broadly used approach for determining the impact of environmental and spatial processes on species composition (Borcard, Legendre & Drapeau, 1992; Gilbert & Lechowicz, 2004; Cottenie, 2005; Van der Gucht et al., 2007; Heino et al., 2015). Here, environment independent of both space and GR ([E|S,GR], fraction **a** in Fig. 3) measures the effect of water physico-chemistry on guild density. Space, independent of both environment and GR ([S|E,GR], fraction **b** in Fig. 3), captures dispersal or historic processes. Competitor/facilitator guild richness (GR) may affect community makeup independently of both environment and space or in conjunction with them (hypotheses 1 and 2). Pure GR ([GR|E,S], fraction **c** in Fig. 3) reflects the independent effect of GR and indicates poor growth in suitable and accessible environments (pure competitor GR term) or sustained growth in unsuitable environments (pure facilitator GR term). Environment-GR covariance ([E∩GR|S], fraction **e** in Fig. 3) will be detected if guild abundance is driven by an environmentally constrained competitor or facilitator, i.e., the environment affects a target guild by providing favorable or unfavorable conditions for its competitor/facilitator. Space-GR covariance ([S∩GR|E], fraction **f** in Fig. 3) implies that the competitor/facilitator is spatially confined. The environment-space-GR covariance ([E∩S∩GR], fraction **g** in Fig. 3) indicates that the influence of the competitor/facilitator is both environmentally and spatially dependent. Finally, the environment-space covariance ([E∩S|GR], fraction **d** in Fig. 3) measures the spatially structured environment.

Consistent with Hypothesis 1, the effect of competitor GR is expected to be strong for the tolerant guild in the shared preference mode, both guilds in the distinct preference mode, and donors in the facilitative mode, while a detectable influence of facilitator GR is projected for the recipient guild in the facilitative mode (Table 1). Consistent with Hypothesis 2, the environment is envisioned to have a differential effect on guilds across interaction modes, i.e., weak on tolerant and donor guilds but prominent otherwise (Table 1). Thus, the proposed framework discriminates the responses of guild abundance to environmental filtering *sensu stricto* (abiotic environment, fraction **a** in Fig. 3) vs. biotic interaction (fraction **c** in Fig. 3) and their covariance (fraction **e** in Fig. 3), which has been a challenge in ecology (Kraft et al., 2015). It further projects what guilds would be subjects of each of these influences based on their characteristic tradeoffs.

The proposed framework was tested with continental data on benthic diatoms, spatial variables, and water chemistry. Representative of the shared preference mode are the low profile (tolerant) and the motile (dominant) guilds shifting along a eutrophication gradient (nitrogen + phosphorus) (Passy, 2007). Low profile species can coexist with motile species but they are inferior competitors under high resource supply (benign environments) due to unfavorable biofilm position in the understory, where access to resources is impeded (Passy & Larson, 2011). In contrast, motile species can move freely within the overstory, but they require high nutrient levels and dominate in eutrophic conditions (Passy, 2007; Soininen et al., 2016). Representative of the distinct preference mode are the acidophilous and alkaliphilous low profile guilds segregating along a pH gradient. Acidophilous and alkaliphilous low profile species generally have low nutrient demands and can coexist at circumneutral pH but their pH optima are in the acidic (at pH < 7) vs. non-acidic range (at pH ≥ 7), respectively (Van Dam, Mertens & Sinkeldam, 1994). The facilitative mode is represented by nitrogen fixers and eutrophic non-nitrogen fixers (motile guild), transitioning along a nitrogen gradient. Nitrogen fixers convert atmospheric N_2_ to usable forms of nitrogen, which can be delivered to the rest of the community, stimulating the non-nitrogen fixing species (Grimm & Petrone, 1997; Agawin et al., 2007; Beversdorf, Miller & McMahon, 2013). However, nitrogen fixers are at a competitive disadvantage at high nitrogen concentrations, where their abundance sharply declines (Stancheva et al., 2013).

The selected gradients of nutrient enrichment and pH are highly influential for diatoms (Soininen, 2007) and generate different opportunities for tradeoff and facilitation, e.g., dominance-tolerance along a nutrient gradient, distinct preferences along a non-depletable, regulatory gradient, such as pH (Wisheu, 1998), and a donor-recipient relationship along a nitrogen gradient.

## Materials and Methods

### Environmental and diatom data

Data on local chemistry and diatom community composition were gathered by the National Water-Quality Assessment (NAWQA) Program (http://water.usgs.gov/nawqa) from 655 distinct stream localities, spanning 23 latitudinal and 53 longitudinal degrees. Diatom samples were collected from the richest-targeted habitats, encompassing hard substrates or macrophytes, between June and August from 1993 to 2009. Depending on habitat, a defined area of 25 cobbles, five woody snags or five macrophyte beds was sampled within a stream reach and composited. For details on sampling protocols, visit https://pubs.usgs.gov/of/2002/ofr-02-150/pdf/ofr02-150.pdf. Although diatoms were collected over a 16-year period, the dependent variables (guild densities) had generally non-significant or weak temporal trends, which did not change appreciably the magnitude of the response of these variables to the studied gradients. Stations were sampled for nitrite + nitrate (referred to as nitrogen henceforth), phosphate, pH, water temperature, specific conductance, and dissolved oxygen one to multiple times between 1993 and 2011 and the average of these values was taken. Data on elevation were also available for all sites.

Diatom taxa were identified primarily to species in counts of 590 to 700 cells. Four groups were selected based on traits defining pH preference, conferring resistance to nutrient limitation, or aiding in nutrient uptake. These groups were as follows: acidophilous (acidophilous and acidobiontic species in Van Dam, Mertens & Sinkeldam (1994)), nitrogen fixers (the genera *Denticula*, *Epithemia*, and *Rhopalodia*), and members of the low profile and the motile guild, as defined in Passy (2007) and Rimet & Bouchez (2012). Only two diatom guilds were excluded from this analysis—planktonic and high profile. Planktonic species inhabit the water column and are observed only occasionally in the benthos. High profile species have inconsistent behavior along nutrient gradients and, unlike the low profile and the motile guilds, are not recommended for studying community responses to eutrophication (Soininen et al., 2016). Thus, this investigation encompasses the majority of diatom groups, which are referred to as guilds for simplicity.

The acidophilous guild (69 species of various growth morphologies) has preference for pH <  7, while the other three guilds prefer pH ≥ 7; the nitrogen fixers (13 species) have cyanobacterial endosymbionts that can fix atmospheric nitrogen; the low profile guild (103 species) can tolerate low nutrient supply; and the motile guild (458 species) is eutrophic, sensitive to nutrient limitation (Van Dam, Mertens & Sinkeldam, 1994; Passy, 2007). Total density (cells cm^−2^) and number of species in each guild were recorded for each sample.

**Figure 4:**
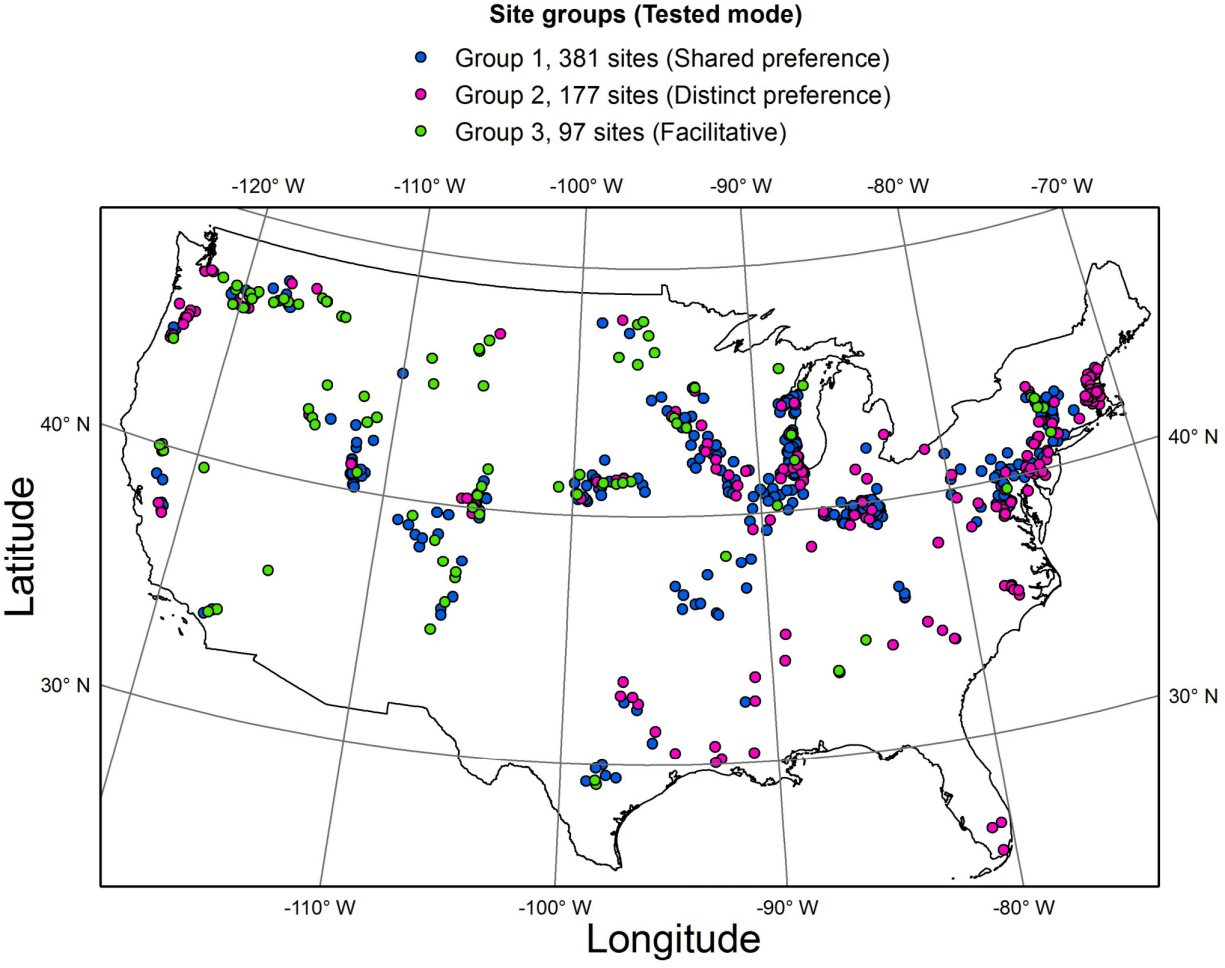
Map of the study area showing the three groups of sites where the three interaction modes were tested.

To test the two hypotheses (Table 1, Fig. 2), the whole dataset was subdivided into three groups, based on the distribution of the four selected guilds along three major gradients (Fig. 4, Table S1). Specifically, low profile and motile guilds (but no acidophilous or nitrogen fixing forms) were found along a eutrophication gradient in group 1 (381 streams). Low profile and acidophilous species (but no nitrogen fixers) were observed along a pH gradient in group 2 (177 streams). Group 3 encompassed streams along a nitrogen gradient with nitrogen fixers and motile species but no acidophilous forms (97 streams). Groups 1, 2 and 3 were used to test the shared preference, distinct preference, and facilitative mode, respectively.

### Statistical analyses

A series of multiple regressions was performed on ln-transformed guild density for each interaction mode using as a predictor set (i) the linear and quadratic terms of the selected environmental variable(s), i.e., pH, ln-transformed PO_4_, and/or NO_2_ + NO_3_, (ii) the linear and quadratic terms of the projected latitude (X) and longitude (Y), or (iii) the linear and quadratic terms of species richness of a competitor/facilitator guild. A stepping procedure was implemented to select only significant predictors. A follow-up variance partitioning using the significant predictors from the aforementioned regressions estimated the unique effects (pure environment, pure space, and pure guild richness) as well as the covariance effects of the predictor sets. A spatial polynomial was chosen in variance partitioning because of its simplicity and lack of systematic bias toward over-estimating the spatial effect, reported for other commonly used spatial approaches (Gilbert & Bennett, 2010). To confirm that the spatial and biotic effects were not over-represented due to limited environmental information, variance partitioning of guild density was also performed using other important environmental variables, including water temperature, specific conductance, dissolved oxygen, and elevation (all ln- or ln(*x* + 1)-transformed).

## Results

### Shared preference mode

**Figure 5:**
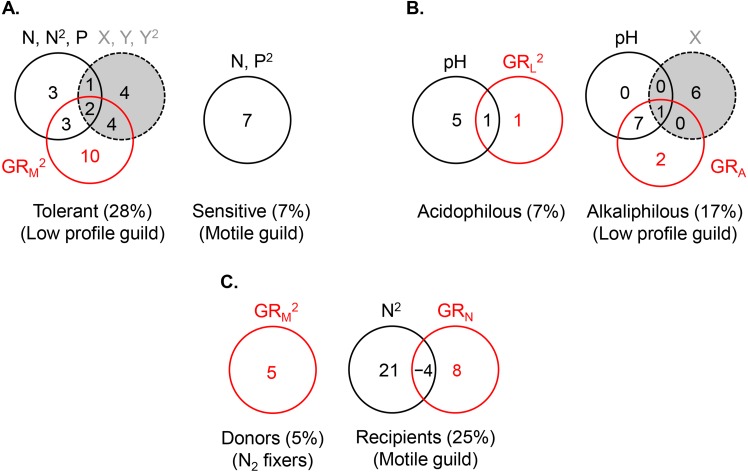
Variance partitioning of ln-transformed guild density (cells cm^−2^) under the shared preference mode, *n* = 381 (A), the distinct preference mode, *n* = 177 (B), and the facilitative mode, *n* = 97**(C)** using a single environmental gradient, i.e., eutrophication (nitrogen and phosphorus) in (A), pH in (B), and nitrogen in (C). The significant terms (linear and/or quadratic) of each predictor are given in the figure. The percentages indicate explained variance and are derived from the respective adjusted *R*^2^. N, nitrite + nitrate; P, phosphate; X, projected latitude; Y, projected longitude; GR_A_, GR_L_, GR_M_, GR_N_, richness of the acidophilous, low profile, motile, and N_2_ fixer guild, respectively; *n*, number of stream localities.

**Figure 6:**
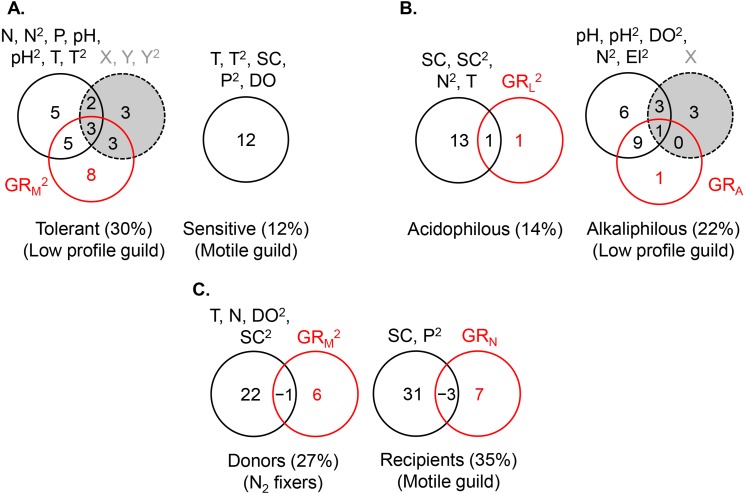
Variance partitioning of ln-transformed guild density (cells cm^−2^) under the shared preference mode, *n* = 381 (A), the distinct preference mode, *n* = 177 (B), and the facilitative mode, *n* = 97 (C) using multiple environmental gradients, including nitrogen (mg L^−1^), phosphorus (mg L^−1^), pH, temperature (° C), specific conductance (µS cm^−1^), dissolved oxygen (mg L^−1^), and site elevation (m). All predictors but pH were ln- or ln(*x* + 1)-transformed. The significant terms (linear and/or quadratic) of each predictor are given in the figure. The percentages indicate explained variance and are derived from the respective adjusted *R*^2^. N, nitrite + nitrate; P, phosphate; SC, specific conductance; DO, dissolved oxygen; T, temperature; El, elevation; X, projected latitude; Y, projected longitude; GR_A_, GR_L_, GR_M_, GR_N_, richness of the acidophilous, low profile, motile, and N_2_ fixer guild, respectively; *n*, number of stream localities. In (B), pH did not enter the acidophilous guild model due to high collinearity with specific conductance (Pearson *r* = 0.73, *P* < 0.00001). In (C), N was significant only in the multivariate model, but not as a single predictor of nitrogen fixer density. Even though in (C) nitrogen had a strong correlation with motile guild density, it did not enter the model due to collinearity with phosphate (Pearson *r* = 0.72, *P* < 0.00001).

The abundance of the low profile guild was most strongly and negatively affected by motile guild richness (GR_M_) (*R*^2^ = 0.20), followed by spatial (*R*^2^ = 0.11) and environmental factors (*R*^2^ = 0.10) (Fig. 5A, the *R*^2^ values represent the total variance explained by each predictor set). The highest density of this guild was observed at the lowest richness of motile species, lowest phosphate concentrations, high latitudes, and extreme longitudes (easternmost and westernmost) (Table 2). Variance partitioning revealed that pure GR_M_, indicative of competition, had the strongest effect on low profile guild density (*R*^2^ = 0.10), while the remaining terms were of lesser importance. Adding other significant environmental variables to the model, i.e., pH and temperature, changed somewhat the fractions of explained variance in the density of this guild but confirmed that competition remained an important contributor (Fig. 6A).

**Table 2:** Statistics of the multiple regressions of ln (guild density) against environmental, spatial, and biotic variables in each interaction mode.

Mode	Guilds	Effect	Coefficient	SE	SC	*t*	*P*-value
**Shared preference**	**Tolerant, low profile guild**	Intercept	10.17	0.53	0.00	19.30	0.00000
	adj. *R*^2^ = 0.28	ln (NO_2_+ NO_3_)	0.17	0.06	0.15	2.99	0.003
	*F* = 22.40, *n* = 381	ln (NO_2_ + NO_3_)^2^	−0.07	0.03	−0.09	−2.05	0.04
		ln (PO_4_)	−0.23	0.07	−0.19	−3.51	0.0005
		Latitude	0.08	0.02	0.16	3.44	0.0007
		Longitude	0.01	0.01	0.08	1.67	0.10^a^
		Longitude^2^	0.001	0.001	0.09	1.76	0.08^a^
		GR${}_{\mathrm{M}}^{2}$	−0.001	0.000	−0.37	−7.37	0.00000
	**Dominant, motile guild**	Intercept	12.43	0.18	0.00	68.02	0.00000
	adj. *R*^2^ = 0.07	ln (NO_2_+ NO_3_)	0.22	0.07	0.18	3.25	0.001
	*F* = 14.26, *n* = 381	ln (PO_4_)^2^	−0.03	0.01	−0.13	−2.35	0.019
**Distinct preference**	**Acidophilous guild**	Intercept	13.21	1.55	0.00	8.51	0.00000
	adj. *R*^2^ = 0.07	pH	−0.70	0.21	−0.24	−3.29	0.001
	*F* = 20.26, *n* = 177	GR${}_{\mathrm{L}}^{2}$	−0.004	0.002	−0.11	−1.48	0.14^c^
	**Alkaliphilous, low profile guild**	Intercept	7.13	2.21	0.00	3.22	0.0015
	adj. *R*^2^ = 0.17	pH	0.34	0.28	0.12	1.22	0.22^b^
	*F* = 13.14, *n* = 177	Latitude	0.08	0.02	0.25	3.70	0.0003
		GR_A_	−0.16	0.07	−0.23	−2.41	0.017
**Facilitative**	**Donor, N**_**2**_**fixer guild**	Intercept	8.35	0.31	0.00	27.26	0.00000
	adj. *R*^2^ = 0.05, *F* = 6.08, *n* = 97	GR${}_{\mathrm{M}}^{2}$	−0.001	0.001	−0.25	−2.47	0.015
	**Recipient, motile guild**	Intercept	10.37	0.44	0.00	23.54	0.00000
	adj. *R*^2^ = 0.25	ln (NO_2_+ NO_3_)^2^	−0.22^d^	0.04	−0.47	−5.23	0.00000
	*F* = 16.79, *n* = 97	GR_N_	0.96	0.29	0.30	3.32	0.0013

**Notes.**

SE standard error SC standardized coefficient GR_A_, GR_L_, GR_M_, GR_N_ richness of the acidophilous, low profile, motile, and N_2_ fixer guild, respectively adj. adjusted *n* number of stream localities

a Variable significant in the regression of ln (guild density) against spatial predictors only.

b Variable significant in the regression of ln (guild density) against environmental predictors only.

c Variable significant in the regression of ln (guild density) against GR${}_{\mathrm{M}}^{2}$ only.

d The lowest values of ln (NO_2_ + NO_3_) are negative, therefore, the highest values of ln (NO_2_ + NO_3_)^2^ are positive and the negative slope indicates that motile guild density increases with ln (NO_2_ + NO_3_).

The density of the dominant motile guild responded only to N and P concentrations (*R*^2^ = 0.07) (Table 2, Fig. 5A). The highest motile guild density was recorded at the highest N levels and intermediate P concentrations. The variance explained by the environment increased when specific conductance, dissolved oxygen, and temperature were included in the model but N and P were stronger predictors of motile guild density (Fig. 6A).

Thus, along a eutrophication and other physico-chemical gradients, the major density drivers included competition for the tolerant guild and the environment for the motile guild (Table 1).

### Distinct preference mode

Only the density of the low profile guild (alkaliphilous) but not of the acidophilous guild was significantly negatively correlated with motile guild richness (the quadratic term, GR${}_{\mathrm{M}}^{2}$). However, residual low profile guild density (after extracting the effect of GR${}_{\mathrm{M}}^{2}$) showed nearly identical responses to pH and acidophilous guild richness as the raw low profile guild density, confirming that the following results were not affected by motile guild richness.

The acidophilous guild density was primarily determined by pH (*R*^2^ = 0.06), while alkaliphilous guild richness (GR_L_) had a weak effect (Fig. 5B). The greatest density of the acidophilous guild was detected at low pH and low GR_L_ (Table 2). The expanded environmental model, including specific conductance, nitrogen, and water temperature, increased the pure environmental fraction but did not alter the pure GR_L_ fraction (Fig. 6B).

The density of the alkaliphilous low profile guild responded most strongly to GR_A_ (*R*^2^ = 0.11), followed by pH (*R*^2^ = 0.09), and latitude (*R*^2^ = 0.07) (Fig. 5B). Alkaliphilous guild density peaked in streams with higher pH, fewer acidophilous taxa, and higher latitude (Table 2). Variance partitioning showed that pH-GR_A_ covariance (*R*^2^ = 0.07) and pure space (*R*^2^ = 0.06) captured the highest proportion of explained variance (Fig. 5B). Adding all significant variables to the environmental model, including pH, dissolved oxygen, elevation, and nitrogen, increased the pure environmental fraction and reduced the pure spatial fraction, but had little effect on the environment-GR_A_ covariance (Fig. 6B). This indicated that an environmentally constrained competitor continued to exhibit the highest impact on the acidophilous guild density and that the spatial effect along the pH gradient was largely due to unmeasured environmental factors.

Thus, acidophilous guild density was primarily driven by pH (or specific conductance), while alkaliphilous guild density, by an environmentally constrained competitor (Table 1).

### Facilitative mode

The density of the low profile guild, which was present in the streams of group 3, had no influence on either donors or recipients. Nitrogen fixer density showed no response to N concentrations but declined significantly with motile guild richness, GR_M_ (*R*^2^ = 0.05, Table 2), as hypothesized. The expanded environmental model, encompassing dissolved oxygen, water temperature, specific conductance, and nitrogen, did not alter appreciably the fraction explained by pure GR_M_ (Fig. 6C) but explained a large proportion of the variance in nitrogen fixer abundance (*R*^2^ = 0.22, Fig. 6C). Space had no effect on either donor or recipient guild density.

The density of the recipient motile guild responded positively to nitrogen concentration and N_2_ fixer richness (GR_N_) (Table 2). Variance partitioning showed that pure nitrogen concentration captured much of the explained variance (*R*^2^ = 0.21), followed by pure N_2_ fixer richness (i.e., facilitation, *R*^2^ = 0.08) (Fig. 5C). Selection of all significant environmental variables, including specific conductance and phosphate, and subsequent variance partitioning indicated that pure GR_N_ remained an important contributor to the explained variance of motile guild density (Fig. 6C).

Thus, along the nitrogen gradient, donor’s abundance was under weak competitive control, whereas recipient’s abundance experienced comparatively strong environmental influence and detectable facilitation. Along multiple physico-chemical gradients, the environmental effect on donors increased but remained weaker than this on recipients, while the competitive effect on donors remained unchanged (Table 1).

## Discussion

### Trait composition and metacommunity variability

A large number of investigations across scales, ecosystems, and organismal groups has shown that environmental and spatial factors constrain species composition in distinct ways, assessed by variance partitioning into fractions explained by pure environment, pure space, and their covariance (Borcard, Legendre & Drapeau, 1992; Potapova & Charles, 2002; Soininen, Paavola & Muotka, 2004; Beisner et al., 2006; Mykrä, Heino & Muotka, 2007). Further research conceptualized the community responses to these factors within the metacommunity framework, relating the fractions of explained variance to particular environment- or dispersal related mechanisms (Gilbert & Lechowicz, 2004; Cottenie, 2005; De Bie et al., 2012; Heino et al., 2015). Notably, the focus of this research has been nearly exclusively on species variability along environmental and spatial gradients. However, recent studies have recognized that the metacommunity patterns and the role of environmental and spatial factors depend on trait composition, e.g., good vs. poor dispersers (Thompson & Townsend, 2006), generalists vs. specialists (Pandit, Kolasa & Cottenie, 2009), common vs. rare species (Siqueira et al., 2012), species with small vs. large propagules or body sizes (Hájek et al., 2011; De Bie et al., 2012), and species across diatom guilds and growth forms (Göthe et al., 2013; Algarte et al., 2014). The present study built on this rich body of literature but further offered a framework that integrated facilitation and resource partitioning theory and predicted changes in a community function, i.e., guild abundance accumulation, along continental environmental and spatial gradients.

Across all interaction modes, detectable relationships were observed between guild abundance and environmental and biotic factors. However, the influence of these factors depended on interaction mode, consistent with both hypotheses (Table 1). With the exception of the low profile guild, spatial variables had no effect on guild abundance.

### Shared preference mode

In the dominance-tolerance mode, the tolerant guild responded comparatively strongly to competitor richness, while the sensitive guild, to the environment, as generally predicted (Table 1). The environment played some role in the abundance variability of the tolerant guild but much of this effect was due to covariance with motile guild richness (GR_M_) and GR_M_ + space. For example, higher N and P levels increased GR_M_ and were associated with a decline in tolerant guild density, possibly as a result of intensified competition. Pure environment increased in importance when guild abundance was examined along multiple gradients but much more so in the dominant than in the tolerant guild. The pure spatial and biotic effects, detected only for the tolerant guild abundance, changed little, indicating that they were not spurious, i.e., due to unmeasured but important environment. Spatial effects were detected in the tolerant low profile guild but not in the dominant motile guild. Disparity in the spatial responses of these guilds was also documented at a much smaller scale (Göthe et al., 2013), where dispersal effects are likely to be much more pronounced. Pandit, Kolasa & Cottenie (2009) reported greater spatial control over generalists but stronger environmental control over specialists, which correspond respectively to the tolerant and dominant guilds here. However, the present study further showed that the biotic influence (competition) was a prominent source of abundance variability in the tolerant generalists. Therefore, biotic impact must be incorporated in metacommunity studies to correctly identify the mechanisms underlying generalists’ abundance.

### Distinct preference mode

The acidophilous and alkaliphilous guild densities showed opposing responses along the pH gradient, consistent with distinct optima. However, the nature of this response was different—the acidophilous guild was constrained primarily by pure pH, while the alkaliphilous guild, by the covariance of pH with competitor richness. This suggests that departure from optimal pH intensified both the physiological and biotic stress on the alkaliphilous guild. The acidophilous guild was under much weaker biotic effect, which may be due to the more diverse growth morphology of this guild. It includes low profile, high profile, and motile forms, allowing more efficient resource utilization compared to the alkaliphilous guild, which comprises only low profile forms. Spatial factors had an impact only on the alkaliphilous guild, which increased in density with latitude. A global study of these guilds too documented significant latitudinal variation only for the alkaliphilous low profile guild (Soininen et al., 2016). Here, this guild was found to be driven by latitudinal variability in dissolved oxygen and elevation, both increasing at higher latitudes. Thus, the environment controlled the density of both guilds, but competitive effects were observed only in the alkaliphilous guild, providing only partial support of the proposed framework. The magnitude of the pure space fraction in the low profile guild model for both the shared preference and the distinct preference mode suggests that dispersal is not an important factor driving the abundance of this guild.

### Facilitative mode

In the facilitative mode, a competitive effect was detected only in donors and a nitrogen effect, only in recipients, as expected (Table 1, Fig. 2C). Nitrogen fixer richness had a positive influence on the abundance of nitrogen-dependent forms, consistent with facilitation. In contrast, recipients had a negative influence on nitrogen fixers, which displayed the lowest abundance at the highest recipient richness in higher nitrogen conditions. These results are supported by experiments with marine phytoplankton demonstrating that the relationship between nitrogen fixers and non-nitrogen fixers switches from facilitation to competition with the increase in nitrogen concentrations (Agawin et al., 2007).

Environmental influences other than nitrogen contributed to the explained variance in guild density but did not affect conspicuously the impact of competition and facilitation. Specifically, nitrogen fixer density increased most strongly with temperature, consistent with previously reported temperature dependence of nitrogen fixation (Marcarelli & Wurtsbaugh, 2006). The density of this guild declined with nitrogen after the effects of other influential variables were accounted for in the full model. These results suggest that at a continental scale, nitrogen fixer density is primarily driven by temperature (*R*^2^ = 0.10), and secondarily, by water chemistry, including nitrogen (*R*^2^ = 0.04–0.05). Motile density responded positively to phosphate and specific conductance, which is expected, considering the eutrophic nature of this guild (Passy, 2007). In the marine environment, nitrogen fixation has controlled primary production at geologic scales (Falkowski, 1997). Here we see that in streams, nitrogen fixer richness may contribute to the abundance accumulation of the most speciose diatom guild, the motile forms, at a continental scale.

### Summary of variance partitioning results across interaction modes

The present results confirmed that environment, competition, and facilitation, as defined in Table 1 and Fig. 3, had an impact on abundance across guilds but the magnitude of this impact was guild-specific, confirming both hypotheses 1 and 2. Space, on the other hand, had no effect on all but the low profile guild. The density of the tolerant guild in the shared preference mode, both guilds in the distinct preference mode, and the donor guild in the facilitative mode declined to a different degree with increasing richness of their competitor. In some cases, this decline could be attributed not only to competitor diversity alone but also to the stimulating effect of the environment on competitor diversity. For the low profile guild density, the geographic dependence of competitor richness also had an effect. The density of nitrogen-dependent forms in the facilitative mode increased with nitrogen fixer richness at stressful, low nitrogen environments, consistent with the stress gradient hypothesis. Therefore, the realized niche of most of the studied guilds was intricately controlled not only by inter-guild interactions but also by environmental and, on occasion, by spatial factors modulating the biotic effect. Considering that the chemical environment and competitor/facilitator guild richness had a much stronger impact on abundance than space, it becomes apparent that factors controlling growth rates overrode dispersal and historic mechanisms. It is worth mentioning that the weak spatial response may be a result of a limitation of the dataset, which was collected over multiple years and thus may have not adequately reflected dispersal effects. Nevertheless, the observed abundance patterns in microorganisms are akin to those of taxonomic composition, which too have been reported to respond more strongly to environmental than spatial factors (Verleyen et al., 2009). Notably, much of the abundance variance across guilds remained unexplained even when multiple environmental gradients were considered. This could be attributed to intra-guild interactions or disturbance, including grazing and scouring, shown to cause a substantial biomass reduction in the periphyton (Hillebrand, 2009).

This study demonstrated that guilds were subjected to different biotic influences, both competitive and facilitative. These influences were disentangled to a large extent by selecting sites, where non-target guilds (i.e., guilds not involved in a particular mode) and their underlying abiotic gradients were either absent or non-consequential. Therefore, metacommunity analyses, which rarely include guild information or perform such site segregation, are likely to encounter several challenges, including: (i) underestimation of the explained variance in species composition or abundance when competition and facilitation have substantial independent effects (Figs. 5 and 6), (ii) incomplete understanding of the role of the environment and space when they co-vary with trait composition (Figs. 5 and 6), and (iii) potential failure to detect an overall environmental effect when strong but opposing environmental influences on guild abundance cancel each other out. Given that competitor/facilitator guild richness contributed to explaining the abundance patterns of target guilds both independently as well as interactively with the environment and occasionally with space, a comprehensive metacommunity modelling needs to be trait-explicit. Such modelling will be of particular interest to conservation because direct environmental influence on a target group vs. indirect environmental influence through a competitor or a facilitator, may require different management strategies.

### Implications for functional biogeography and biodiversity-ecosystem functioning

The proposed framework has important implications for two rapidly growing research areas, i.e., functional biogeography (Violle et al., 2014) and biodiversity-ecosystem functioning (BEF) (Loreau, 2010; Naeem, Duffy & Zavaleta, 2012). Functional biogeography explores the spatial distribution of trait diversity. Biodiversity-ecosystem functioning focuses on how species richness controls ecosystem functions, including biomass production. It posits that greater biodiversity allows complementary resource utilization and accumulation of higher biomass. However, the relationship between biodiversity and biomass or biovolume is non-linear—most often it is saturating (Hooper et al., 2005; Cardinale et al., 2012), while in stream algae, it is unimodal and comparatively weak (Passy & Legendre, 2006). These observations indicate that competition may play an important role at high biodiversity, causing biomass production to taper off or decline, but this role is not well understood. By exploring competitive and facilitative interactions, the present study offers an insight into the biotic constraints on community function and how they are affected by environmental and spatial factors. This approach provides a better understanding of the sources of variability in abundance accumulation and better predictive power in describing this variability than the conventional regression of abundance against biodiversity. For example, in streams from groups 1, 2, and 3, total community richness (the linear and quadratic term) explained, respectively, only 5%, 5%, and 4% of the variance in total density, which is directly proportional to biovolume (a measure of biomass). Alternatively, the regression models in Table 2, including environmental and spatial predictors as well as competitor/facilitator richness, captured 7–28%, 7–17%, and 5–25% of the variance in guild density in groups 1, 2, and 3, respectively. Moreover, the variance partitioning models (Figs. 5 and 6) indicated that different abiotic and biotic processes generated the abundance patterns across guilds. It is recommended that future BEF studies deconstruct the richness-biomass relationship and explore how biomass of target guilds is defined by their own richness as well as richness of competitor and facilitator guilds and environmental and spatial factors to gain a better understanding of the drivers of biomass production. Clearly, the proposed framework, elucidating the complex and intertwined guild responses to abiotic and biotic factors, has the potential to further our knowledge of the biodiversity-ecosystem functioning and the causes of geographic variability in traits.

##  Supplemental Information

10.7717/peerj.3885/supp-1 Supplemental Information 1 Species data Guild affiliation, absolute, and relative abundance for all species in each of the studied guilds across the three groups where the shared preference (Table 1), the distinct preference (Table 2), and the facilitative mode (Table 3) were tested.

10.7717/peerj.3885/supp-2 Supplemental Information 2 Sample data Spatial, environmental, and biotic data for samples in groups where the shared preference (Table 1), the distinct preference (Table 2), and the facilitative mode (Table 3) were tested. For each sample, the sample ID, station ID, study unit, and time of collection are also provided.

10.7717/peerj.3885/supp-3 Supplemental Information 3 Table S1 Water chemistry variability across the three studied groups of streams, where the shared preference (group 1), the distinct preference (group 2), and the facilitative mode (group 3) were tested. The concentrations of NO_2_ + NO_3_ and PO_4_ were measured in mg L^−1^. SD = standard deviation; and *n*= number of stream localities. A different letter in the superscript indicates significant differences (*P* < 0.05) in parameter values (ln-transformed in the case of NO_2_ + NO_3_ and PO_4_) across the three groups, following ANOVA and Tukey post-hoc test.

## Additional Information and Declarations

### Data Availability

The following information was supplied regarding data availability: The raw data have been supplied as a Supplementary File.

## References

[ref-1] Agawin NSR, Rabouille S, Veldhuis MJW, Servatius L, Hol S, Van Overzee HMJ, Huisman J (2007). Competition and facilitation between unicellular nitrogen-fixing cyanobacteria and non-nitrogen-fixing phytoplankton species. Limnology and Oceanography.

[ref-2] Algarte VM, Rodrigues L, Landeiro VL, Siqueira T, Bini LM (2014). Variance partitioning of deconstructed periphyton communities: does the use of biological traits matter?. Hydrobiologia.

[ref-3] Beisner BE, Peres PR, Lindstrom ES, Barnett A, Longhi ML (2006). The role of environmental and spatial processes in structuring lake communities from bacteria to fish. Ecology.

[ref-4] Bertness MD, Callaway R (1994). Positive interactions in communities. Trends in Ecology & Evolution.

[ref-5] Bertness MD, Shumway SW (1993). Competition and facilitation in marsh plants. American Naturalist.

[ref-6] Beversdorf LJ, Miller TR, McMahon KD (2013). The role of nitrogen fixation in cyanobacterial bloom toxicity in a temperate, eutrophic lake. PLOS ONE.

[ref-7] Borcard D, Legendre P, Drapeau P (1992). Partialling out the spatial component of ecological variation. Ecology.

[ref-8] Bruno JF, Stachowicz JJ, Bertness MD (2003). Inclusion of facilitation into ecological theory. Trends in Ecology & Evolution.

[ref-9] Cardinale BJ, Duffy JE, Gonzalez A, Hooper DU, Perrings C, Venail P, Narwani A, Mace GM, Tilman D, Wardle DA, Kinzig AP, Daily GC, Loreau M, Grace JB, Larigauderie A, Srivastava DS, Naeem S (2012). Biodiversity loss and its impact on humanity. Nature.

[ref-10] Cottenie K (2005). Integrating environmental and spatial processes in ecological community dynamics. Ecology Letters.

[ref-11] De Bie T, De Meester L, Brendonck L, Martens K, Goddeeris B, Ercken D, Hampel H, Denys L, Vanhecke L, Van der Gucht K, Van Wichelen J, Vyverman W, Declerck SAJ (2012). Body size and dispersal mode as key traits determining metacommunity structure of aquatic organisms. Ecology Letters.

[ref-12] Falkowski PG (1997). Evolution of the nitrogen cycle and its influence on the biological sequestration of CO_2_ in the ocean. Nature.

[ref-13] Gilbert B, Bennett JR (2010). Partitioning variation in ecological communities: do the numbers add up?. Journal of Applied Ecology.

[ref-14] Gilbert B, Lechowicz MJ (2004). Neutrality, niches, and dispersal in a temperate forest understory. Proceedings of the National Academy of Sciences of the United States of America.

[ref-15] Göthe E, Angeler DG, Gottschalk S, Lofgren S, Sandin L (2013). The influence of environmental, biotic and spatial factors on diatom metacommunity structure in Swedish headwater streams. PLOS One.

[ref-16] Grimm NB, Petrone KC (1997). Nitrogen fixation in a desert stream ecosystem. Biogeochemistry.

[ref-17] Hájek M, Rolecek J, Cottenie K, Kintrová K, Horsák M, Poulicková A, Hájková P, Fránková M, Díte D (2011). Environmental and spatial controls of biotic assemblages in a discrete semi-terrestrial habitat: comparison of organisms with different dispersal abilities sampled in the same plots. Journal of Biogeography.

[ref-18] He Q, Bertness MD (2014). Extreme stresses, niches, and positive species interactions along stress gradients. Ecology.

[ref-19] He Q, Bertness MD, Altieri AH (2013). Global shifts towards positive species interactions with increasing environmental stress. Ecology Letters.

[ref-20] Heino J, Melo AS, Siqueira T, Soininen J, Valanko S, Bini LM (2015). Metacommunity organisation, spatial extent and dispersal in aquatic systems: patterns, processes and prospects. Freshwater Biology.

[ref-21] Hillebrand H (2009). Meta-analysis of grazer control of periphyton biomass across aquatic ecosystems. Journal of Phycology.

[ref-22] Hooper DU, Chapin FS, Ewel JJ, Hector A, Inchausti P, Lavorel S, Lawton JH, Lodge DM, Loreau M, Naeem S, Schmid B, Setala H, Symstad AJ, Vandermeer J, Wardle DA (2005). Effects of biodiversity on ecosystem functioning: a consensus of current knowledge. Ecological Monographs.

[ref-23] Jamoneau A, Passy SI, Soininen J, Leboucher T, Tison-Rosebery J (2017). Beta diversity of diatom species and ecological guilds: Response to environmental and spatial mechanisms along the stream watercourse. Freshwater Biology.

[ref-24] Kraft NJB, Adler PB, Godoy O, James EC, Fuller S, Levine JM (2015). Community assembly, coexistence and the environmental filtering metaphor. Functional Ecology.

[ref-25] Loreau M (2010). Linking biodiversity and ecosystems: towards a unifying ecological theory. Philosophical Transactions of the Royal Society B: Biological Sciences.

[ref-26] Marcarelli AM, Wurtsbaugh WA (2006). Temperature and nutrient supply interact to control nitrogen fixation in oligotrophic streams: an experimental examination. Limnology and Oceanography.

[ref-27] McGill BJ, Enquist BJ, Weiher E, Westoby M (2006). Rebuilding community ecology from functional traits. Trends in Ecology & Evolution.

[ref-28] Mykrä H, Heino J, Muotka T (2007). Scale-related patterns in the spatial and environmental components of stream macroinvertebrate assemblage variation. Global Ecology and Biogeography.

[ref-29] Naeem S, Duffy JE, Zavaleta E (2012). The functions of biological diversity in an age of extinction. Science.

[ref-30] Pandit SN, Kolasa J, Cottenie K (2009). Contrasts between habitat generalists and specialists: an empirical extension to the basic metacommunity framework. Ecology.

[ref-31] Passy SI (2007). Diatom ecological guilds display distinct and predictable behavior along nutrient and disturbance gradients in running waters. Aquatic Botany.

[ref-32] Passy SI, Larson CA (2011). Succession in stream biofilms is an environmentally-driven gradient of stress tolerance. Microbial Ecology.

[ref-33] Passy SI, Legendre P (2006). Are algal communities driven toward maximum biomass?. Proceedings of the Royal Society B-Biological Sciences.

[ref-34] Potapova MG, Charles DF (2002). Benthic diatoms in USA rivers: distributions along spatial and environmental gradients. Journal of Biogeography.

[ref-35] Rimet F, Bouchez A (2012). Life-forms, cell-size and ecological guilds of freshwater diatoms. Knowledge and Management of Aquatic Ecosystems.

[ref-36] Rosenzweig ML (1991). Habitat selection and population interactions: the search for mechanism. American Naturalist.

[ref-37] Shurin JB, Allen EG (2001). Effects of competition, predation, and dispersal on species richness at local and regional scales. American Naturalist.

[ref-38] Siqueira T, Bini LM, Roque FO, Couceiro SRM, Trivinho-Strixino S, Cottenie K (2012). Common and rare species respond to similar niche processes in macroinvertebrate metacommunities. Ecography.

[ref-39] Soininen J (2007). Environmental and spatial control of freshwater diatoms—a review. Diatom Research.

[ref-40] Soininen J, Jamoneau A, Rosebery J, Passy SI (2016). Global patterns and drivers of species and trait composition in diatoms. Global Ecology and Biogeography.

[ref-41] Soininen J, Paavola R, Muotka T (2004). Benthic diatom communities in boreal streams: community structure in relation to environmental and spatial gradients. Ecography.

[ref-42] Stancheva R, Sheath RG, Read BA, McArthur KD, Schroepfer C, Kociolek JP, Fetscher AE (2013). Nitrogen-fixing cyanobacteria (free-living and diatom endosymbionts): their use in southern California stream bioassessment. Hydrobiologia.

[ref-43] Thompson R, Townsend C (2006). A truce with neutral theory: local deterministic factors, species traits and dispersal limitation together determine patterns of diversity in stream invertebrates. Journal of Animal Ecology.

[ref-44] Van Dam H, Mertens A, Sinkeldam J (1994). A coded checklist and ecological indicator values of freshwater diatoms from the Netherlands. Netherlands Journal of Aquatic Ecology.

[ref-45] Van der Gucht K, Cottenie K, Muylaert K, Vloemans N, Cousin S, Declerck S, Jeppesen E, Conde-Porcuna JM, Schwenk K, Zwart G, Degans H, Vyverman W, De Meester L (2007). The power of species sorting: local factors drive bacterial community composition over a wide range of spatial scales. Proceedings of the National Academy of Sciences of the United States of America.

[ref-46] Verleyen E, Vyverman W, Sterken M, Hodgson DA, De Wever A, Juggins S, Van de Vijver B, Jones VJ, Vanormelingen P, Roberts D, Flower R, Kilroy C, Souffreau C, Sabbe K (2009). The importance of dispersal related and local factors in shaping the taxonomic structure of diatom metacommunities. Oikos.

[ref-47] Violle C, Reich PB, Pacala SW, Enquist BJ, Kattge J (2014). The emergence and promise of functional biogeography. Proceedings of the National Academy of Sciences of the United States of America.

[ref-48] Wisheu IC (1998). How organisms partition habitats: different types of community organization can produce identical patterns. Oikos.

