# Supplementary material for: Framework for community functioning: synthesis of stress gradient and resource partitioning concepts
Source: PeerJ. 2017 Oct 2;5:e3885. doi: 10.7717/peerj.3885 (PMC5628606; doi:10.7717/peerj.3885)
Supplement: Supplemental Information 3 — Water chemistry variability across the three studied groups of streams, where the shared preference (group 1), the distinct preference (group 2), and the facilitative mode (group 3) were tested. The concentrations of NO2 + NO3 and PO4 were measured in mg L−1. SD = standard deviation; and n= number of stream localities. A different letter in the superscript indicates significant differences (P < 0.05) in parameter values (ln-transformed in the case of NO2 + NO3 and PO4) across the three groups, following ANOVA and Tukey post-hoc test. [file peerj-05-3885-s003.docx]

**Supplemental Table S1.** Water chemistry variability across the three studied groups of streams, where the shared preference (group 1), the distinct preference (group 2), and the facilitative mode (group 3) were tested. The concentrations of NO_2_ + NO_3_ and PO_4_ were measured in mg·L^−1^. SD = standard deviation; and *n* = number of stream localities. A different letter in the superscript indicates significant differences (*P* < 0.05) in parameter values (ln-transformed in the case of NO_2_ + NO_3_ and PO_4_) across the three groups, following ANOVA and Tukey post-hoc test.

| **Group** | **Parameter** | **Minimum** | **Maximum** | **Range** | **Median** | **Mean** | **SD** |
| --- | --- | --- | --- | --- | --- | --- | --- |
| 1 (n = 381) | pH^a^ | 6.66 | 9.2 | 2.54 | 7.98 | 7.94 | 0.34 |
|  | NO_2_ + NO_3_^a^ | 0.05 | 12.94 | 12.89 | 1.35 | 2.38 | 2.57 |
|  | PO_4_^a^ | 0.01 | 2.8 | 2.79 | 0.03 | 0.11 | 0.27 |
| 2 (n = 177) | pH^b^ | 5.88 | 8.7 | 2.82 | 7.37 | 7.36 | 0.59 |
|  | NO_2_ + NO_3_^b^ | 0.02 | 9.65 | 9.64 | 0.61 | 1.66 | 2.26 |
|  | PO_4_^b^ | 0.003 | 1.23 | 1.23 | 0.02 | 0.07 | 0.16 |
| 3 (n = 97) | pH^c^ | 7.14 | 8.89 | 1.76 | 8.09 | 8.08 | 0.33 |
|  | NO_2_ + NO_3_^c^ | 0.01 | 9.27 | 9.26 | 0.33 | 0.95 | 1.62 |
|  | PO_4_^b^ | 0.003 | 2.08 | 2.07 | 0.02 | 0.09 | 0.24 |

Streams in group 1, spanning phosphate and nitrite + nitrate gradients, included low profile and motile guilds but no acidophilous or nitrogen fixing forms. Acidophilous and alkaliphilous low profile guilds were examined along a pH gradient in streams from group 2. Motile species are ubiquitous and could not be excluded from group 2, but their effect, which was significant only on the low profile guild density, did not confound the studied relationships of low profile guild density with acidophilous guild richness and pH (described in Results). Nitrogen fixers and motile species but no acidophilous forms were detected in group 3, spanning a nitrite + nitrate gradient. Low profile species had no impact on either response variable (density of nitrogen fixing and motile guilds). Removal of samples with acidophilous species from groups 1 and 3 ensured that the effect of this group was eliminated and this of pH, minimized. For example, both group 1 and 3 had significantly higher pH than group 2 (Supplemental Table S1). Removal of samples with nitrogen fixers from groups 1 and 2 eliminated any potential confounding effect of nitrogen facilitation. Group 1, with the highest mean nitrogen and phosphate levels, encompassed oligotrophic to eutrophic sites; group 2, with the lowest mean pH, included acidified and non-acidified sites of lower nutrient concentrations; and group 3, with the lowest mean nitrogen, included oligotrophic to eutrophic sites as well (Supplemental Table S1).
